# Bandage Contact Lens Application Reduces Fibrotic Wound Healing of Flap Margins after FS-LASIK: A Prospective Randomized Clinical Trial

**DOI:** 10.1155/2019/3074659

**Published:** 2019-01-10

**Authors:** Li-Quan Zhao, Liang-Mao Li, Jun Liu, Peng Li

**Affiliations:** ^1^Department of Ophthalmology, Pudong New Area People's Hospital Affiliated to Shanghai University of Medicine and Health Sciences, Shanghai, China; ^2^Department of Ophthalmology, No. 181 Hospital of the PLA, Guilin, Guangxi, China

## Abstract

**Purpose:**

To assess the efficacy of applying bandage contact lens (BCL) in reducing the fibrotic healing response of flap margins following femtosecond laser in situ keratomileusis (FS-LASIK).

**Methods:**

In this prospective, randomized, interventional, observer-masked trial, 41 patients (82 eyes) with myopia and/or myopic astigmatism were scheduled to undergo FS-LASIK. After surgery, patients were fitted with a BCL in one eye (BCL eyes, *n*=41) but not in the contralateral eye (control eyes, *n*=41), following randomized allocation of the BCL to the left or right eye of each patient. The BCL was left in place overnight and removed the following morning. All eyes subsequently received standardized postoperative treatments. Patients were followed up for 6 months. We evaluated patients' self-reported postoperative symptom scores for pain, photophobia, tearing, and foreign-body sensation. At 6 months after surgery, we examined the corneal flap margin and adjacent regions, and photographed them using slit-lamp biomicroscopy, to subjectively evaluate the wound healing response.

**Results:**

Postoperative pain and photophobia were milder in the BCL group than in the control group (*P*=0.041 and *P*=0.003, respectively), but patients felt more foreign-body sensation in the eye with a BCL than in the control eye (*P*=0.001). There was no significant difference in tearing score between BCL eyes and control eyes (*P*=0.118). Regarding the fibrotic healing response of the flap margin, control eyes showed a wide, bright peripheral circumferential band with a spiculated edge and high reflectivity; conversely, BCL eyes showed a markedly narrower and smoother peripheral circumferential band, with a less spiculated edge and lower reflectivity (*P* < 0.001).

**Conclusion:**

Patients felt less discomfort in eyes treated with a BCL after FS-LASIK than in control eyes. BCL-treated eyes also had a less intense wound healing response at the flap margins than control eyes in some of patients. BCLs may merit consideration as a treatment option after FS-LASIK for special patients. This trial is registered with ChiCTR1800016579.

## 1. Introduction

The use of a femtosecond laser to create a corneal flap is increasingly relied upon in refractive surgery. Using a femtosecond laser rather than a microkeratome for laser-assisted in situ keratomileusis (LASIK) flap creation has several possible advantages, including improved safety, better flap uniformity, and increased predictability of flap thickness, along with a decrease in complications such as buttonhole flaps, partial flaps, free caps, and epithelial defects [1].

However, surgeons may encounter a white, reflective circumferential band of the flap margin using slit-lamp biomicroscopy after femtosecond laser-assisted in situ keratomileusis (FS-LASIK). Many studies have noted that FS-LASIK leaves a much more visible scar at the flap edge than a mechanical microkeratome leaves [2–6]. Furthermore, fibrotic wound healing at the flap margin is associated with corneal inflammation induced by the femtosecond laser, which differs from the reaction to the mechanical microkeratome. Other than medication for alleviating postoperative inflammation, no treatment has been recommended to date to prevent or alleviate the wound healing response. However, whether this flap complication could affect the clinical outcome, including postoperative visual acuity and refraction is unknown.

In our clinical work, we occasionally observe corneal epithelial loosening or epithelial defects during FS-LASIK surgery. In such cases, we apply bandage contact lenses (BCLs) immediately after surgery. In addition to our patients reporting relief of postoperative pain, as well as observing promotion of corneal epithelialization, we also found that the circular scars of flap margins in eyes fitted with a BCL were narrower and less reflective than those without BCLs (unpublished observations).

Many studies have confirmed the therapeutic effects of BCLs after corneal refractive surgery in alleviating discomfort, facilitating healing by re-epithelialization, promoting visual recovery, and decreasing the risk of postoperative infection [7–9]. BCLs are also used to keep the flap in its proper position after LASIK [10].

However, to our knowledge, no studies have investigated whether BCL application can alleviate the healing response of the flap margins after FS-LASIK. Our preliminary study about BCLs focused on the corneal epithelialization, not on the flap margins, and this retrospective study could not draw a firm conclusion. Therefore, we conducted a prospective, randomized controlled study to evaluate the performance of BCLs by assessing subjective symptoms and slit-lamp findings.

## 2. Materials and Methods

### 2.1. Patients

Included in this prospective, interventional, randomized, observer-masked study were 45 patients (90 eyes) scheduled to have FS-LASIK for correction of myopia and/or myopic astigmatism. These cases were treated at the outpatient clinic of the Department of Ophthalmology at the No. 181 Hospital of the PLA, China, from September 2016 to July 2017.

We obtained written informed consent for the procedures from each patient and institutional review board/ethics committee approval from the Institutional Review Board of the No. 181 Hospital of the of PLA. The study protocol followed the guidelines of the Declaration of Helsinki.

Eligibility criteria for both procedures were that patients must be 18 years of age or older, must receive a routine ophthalmic examination, and have a stable refractive error with a minimum calculated residual corneal stromal bed thickness of at least 280 *μ*m.

Patients who had unstable refraction, previous ocular surgery (refractive or other surgical procedures), suspected keratoconus, ocular disease, or systemic disease that might alter the wound healing process (such as diabetes or connective tissue disorders) were excluded from the study.

All the patients included in the study underwent a series of preoperative ophthalmologic examinations, which included measurements of manifest and cycloplegic refraction, uncorrected visual acuity (UCVA), best spectacle-corrected visual acuity (BCVA) (using a Snellen chart and Nidek ACP 8 auto chart projector), slit-lamp biomicroscopy, intraocular pressure (IOP) with noncontact tonometry (CT-80, Topcon), corneal pachymetry (SP-3000 pachymeter, TOMEY Laboratories, Inc.), corneal topography (Orbscan II, Bausch and Lomb), and fundoscopy.

All patients received bilateral FS-LASIK surgery. A BCL was placed in one eye immediately after surgery, and no treatment was applied to the contralateral eye, which served as an internal control.

### 2.2. Randomization

A dynamic allocation scheme was used to create an even number of blocks. Simple randomization was then used to allocate BCL placement to the right or left eye of each patient. A sealed envelope with the assigned treatment option was used by an independent observer as a record of patient allocation. The surgeons and patients were blinded to the group allocations before surgery.

### 2.3. Surgery

All surgical treatments were performed by the same experienced surgeon (Zhao LQ).

The VisuMax femtosecond laser system (Carl Zeiss Meditec AG, Germany) was used at a repetition rate of 500 kHz and a pulse energy of 140 nJ to create the flaps. The flaps had a diameter of 7.9 mm, with standard 90-degree hinges and 90-degree side-cut angles. A laser spot separation of 4.5 *μ*m was set for the lamellar flap cut and 2.0 *μ*m for the side cut. The target flap thickness was 120 *μ*m. The hinges were set in a superior orientation with a hinge width of 3.8 mm. Stromal tissue ablation was performed with the MEL 80 (Carl Zeiss Meditec AG, Germany) excimer laser at a repetition rate of 250 kHz, a pulse energy of 150 nJ, and an optical zone size of 5.75–6.50 mm using tissue-saving or Aberration Smart Ablation. Each patient had the same optical zone and ablation profile in both eyes.

After ablation, balanced salt solution was instilled to rinse the stromal bed and the ocular surface. The flap was repositioned and stretched using surgical sponges, and care was taken to maintain the uniformity of the flap margins. The corneal surface was dried with a sponge. Finally, each patient received a sterile soft silicone-hydrogel BCL (PureVision, Bausch and Lomb, USA) placed onto the cornea of one eye, followed by intraoperative application of topical tobramycin 3 mg/mL and dexamethasone 1 mg/mL. The positioning and fit of the lens were examined under a slit lamp.

### 2.4. Postoperative Follow-Up

All eyes received standardized postoperative treatment.

The BCLs of the intervention group were removed one day after surgery. Postoperative medications included tobramycin 3 mg/mL and dexamethasone 1 mg/mL drops applied four times daily for the first week. The steroid drops were then tapered over the subsequent 3 weeks as follows: fluorometholone 0.1% drops three times daily on days 8–14 postoperatively, twice daily on days 15–21, and once daily on days 22–28. A preservative-free tear supplement (sodium hyaluronate eye drops) was given as needed for the first few months.

Follow-up examinations were scheduled at 1 day, 3 days, 1 week, and 1, 3, and 6 months after surgery. Postoperative 1 day, 3 days, and 6 months UCVA, postoperative 6 months BCVA, refraction, and IOP were assessed.

The patients were evaluated for subjective symptoms using a standard questionnaire on the first day of follow-up [11]. The questionnaire included questions regarding postoperative symptoms such as pain, photophobia, foreign-body sensation, and tearing. Each symptom was graded on a scale of 0–3, where 0 indicated no complaints and 1, 2, and 3 indicated mild, moderate, and severe complaints, respectively.

At the last follow-up visit, 6 months postoperatively, the flap margin and adjacent regions were examined using slit-lamp biomicroscopy. All images were acquired by the same technician (Li P.), who was blinded to the assigned treatments. The images were reviewed by two independent observers (Li L. M. and Liu J.), who were also blinded to group assignments. The evaluation criteria were based on the relative width, regularity, and high or low reflectivity of the flap margin, which are indicators of its healing response. The assessment consisted of comparing the BCL-treated eye with the non-BCL–treated eye using these criteria. The eye with the smoother, narrower, and less reflective flap-margin circumferential band was identified; otherwise no difference between the two eyes was reported. Discordance between observers regarding these comparisons was resolved through discussion until 100% agreement was reached.

### 2.5. Statistical Analysis

A statistical software package (SPSS 17.0) was used for all analyses. Numerical data are presented as the mean ± standard deviation and categorical data as percentages. An analysis of variance was performed to test for significant differences between BCL and control eyes in preoperative and postoperative refraction, UCVA, BCVA, and IOP; central corneal thickness (CCT); ablation depth; postoperative subjective symptoms; and flap-margin healing. A paired *t*-test or the Wilcoxon signed-rank test was used, and *P* ≤ 0.05 was considered statistically significant.

## 3. Results

### 3.1. Patient Characteristics

Forty-six patients were enrolled in this randomized controlled trial. One patient did not tolerate the BCL the night after surgery and was given emergency treatment after removing the BCL. In two patients, the slit-lamp examination 1 day after surgery revealed that the BCL had fallen out by itself, and the patients did not know when it fell out. Two patients dropped out, one at 1 month and one at 3 months postoperatively. In the end, 82 eyes of the 41 patients were analyzed.

The clinical characteristics of BCL eyes and control eyes are listed in https://www.ncbi.nlm.nih.gov/pmc/articles/PMC3340826/table/ijo-04-03-314-t03/ (Table 1). There were no significant differences in preoperative visual acuity, refraction, IOP, or corneal pachymetry between the two groups. There were also no significant differences in surgical parameters between BCL and control eyes, including ablation depth and optical zone.

On the first postoperative day, BCL eyes had worse UCVA than those in the control group. The UCVA of 12 BCL eyes (29.3%) and three control eyes (7.3%) was below 0.8. However, there was no significant difference between the two groups in UCVA improvement 3 days postoperatively. In addition, there was no significant difference between the two groups in UCVA, BCVA, refraction, or IOP at 6 months postoperatively.

### 3.2. Subjective Symptom Relief

Of all included patients, 22 (53.7%) felt less pain in the BCL eye than in the control eye. Six (14.6%) reported no difference between the two eyes in terms of the quantity of pain they felt, whereas 13 (31.7%) felt more pain in the BCL-treated eye than in the untreated eye (*P*=0.040).

Thirty-one patients (75.6%) felt more foreign-body sensation in the BCL eye than in the control eye. Two patients (4.9%) reported no difference in foreign-body sensation between the treated and untreated eye. Eight patients (19.5%) felt less foreign-body sensation in the BCL-treated eye than in the untreated eye (*P*=0.001).

For photophobia, 13 patients (31.7%) reported experiencing less in the BCL eye than in the control eye. Twenty-six patients (63.4%) reported no difference in photophobia between eyes, and only two patients (4.9%) experienced more photophobia in the BCL eye than in the control eye (*P*=0.003).

Twenty-one patients (51.2%) reported less tear production in the BCL eye than in the control eye. Eight (19.5%) reported no difference in tear production between the two eyes. Twelve patients (29.3%) reported that the BCL-treated eye had more tear production than the untreated eye (*P*=0.118) (https://www.ncbi.nlm.nih.gov/pmc/articles/PMC4651876/table/ijo-08-06-1131-t02/) (Table 2).

### 3.3. Flap-Margin Healing

Throughout the study, no dislocation of the LASIK flaps was observed.

**Figure 1:**
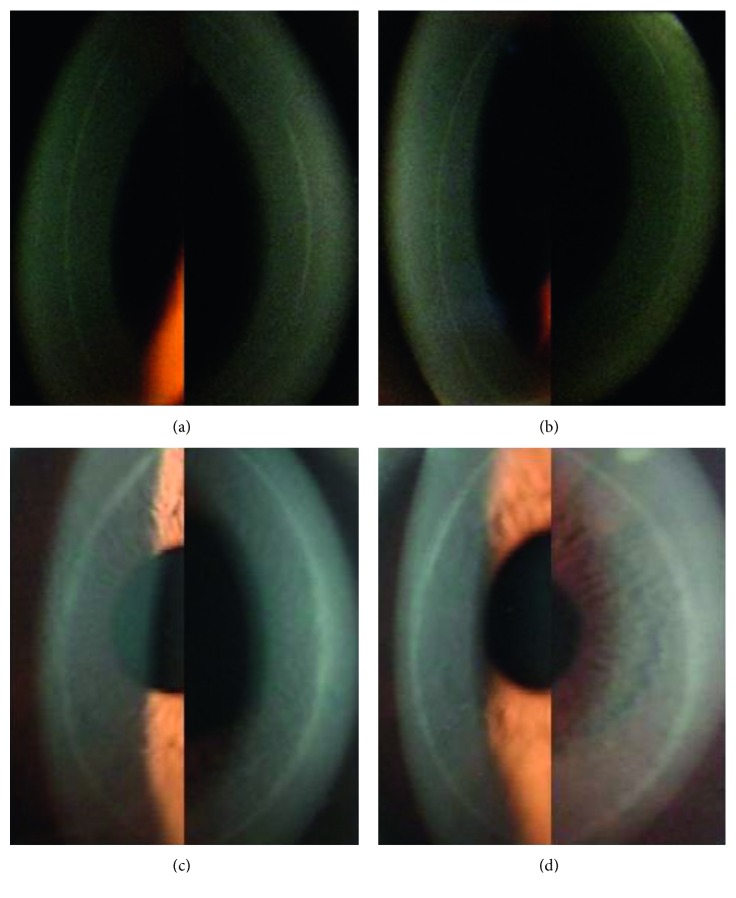
Corneal flap margin and adjacent regions photographed by slit-lamp biomicroscopy at 6 months after surgery. Smooth, narrow, moderately reflective circumferential bands in the control right eye (a) and the BCL-treated left eye (b) of a patient. Smooth, slightly wider circumferential bands in the BCL-treated right eye (c) and the non-BCL-treated left eye (d) of a patient.

**Figure 2:**
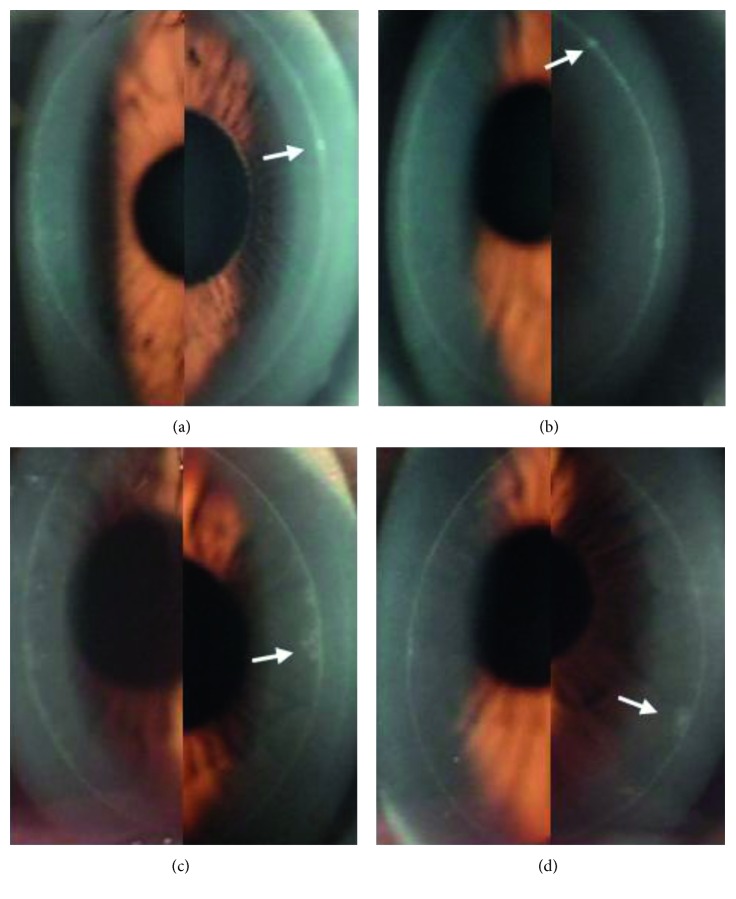
Corneal flap margin and adjacent regions photographed by slit-lamp biomicroscopy at 6 months after surgery. A smooth circumferential band with a small, highly reflective focal scar (white arrow) on the nasal side in the non-BCL-treated right eye (a) and one with a similar scar (white arrow) on the temporal side in the BCL-treated left eye (b) of a patient. A narrow circumferential band with a partially spiculated edge (white arrow) on the nasal side in the non-BCL-treated right eye (c) and one with a partially spiculated temporal side (white arrow) in the BCL-treated left eye (d) of a patient.

At 6 months postoperatively, 18 patients (43.9%) showed no difference in healing response between the BCL and control eyes. Twelve patients had narrow, smooth, and moderately reflective circumferential bands in both eyes (Figures 1(a) and 1(b)). Three patients had a smooth, moderately wide circumferential band in both eyes (Figure 1(c) and 1(d)). One patient had a smooth circumferential band but with a slightly more reflective focal scar in both eyes (Figures 2(a) and 2(b)). Two patients had a narrow circumferential band with a slightly spiculated edge in each eye (Figure 2(c) and 2(d)).

**Figure 3:**
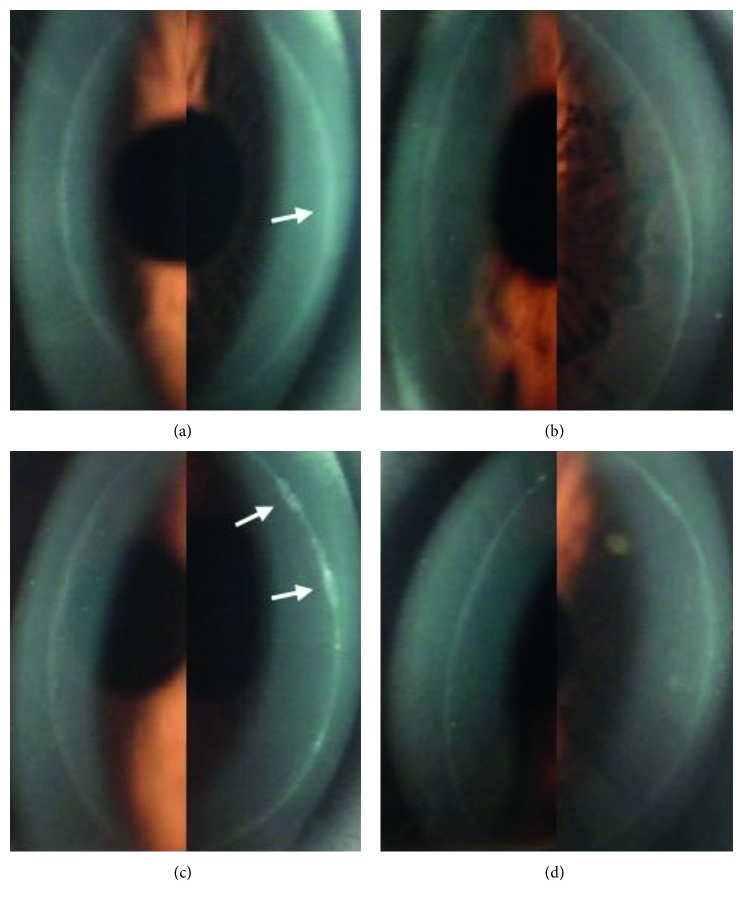
Corneal flap margin and adjacent regions photographed by slit-lamp biomicroscopy at 6 months after surgery. A smooth circumferential band that is slightly wider (white arrow) on the nasal side in the non-BCL-treated right eye (a) and a smooth, narrow circumferential band in the BCL-treated left eye (b) of a patient. A circumferential band with a slightly more reflective focal scar (white arrow) on the nasal side in the non-BCL-treated right eye (c) and a less reflective circumferential band in the BCL-treated left eye (d) of a patient.

**Figure 4:**
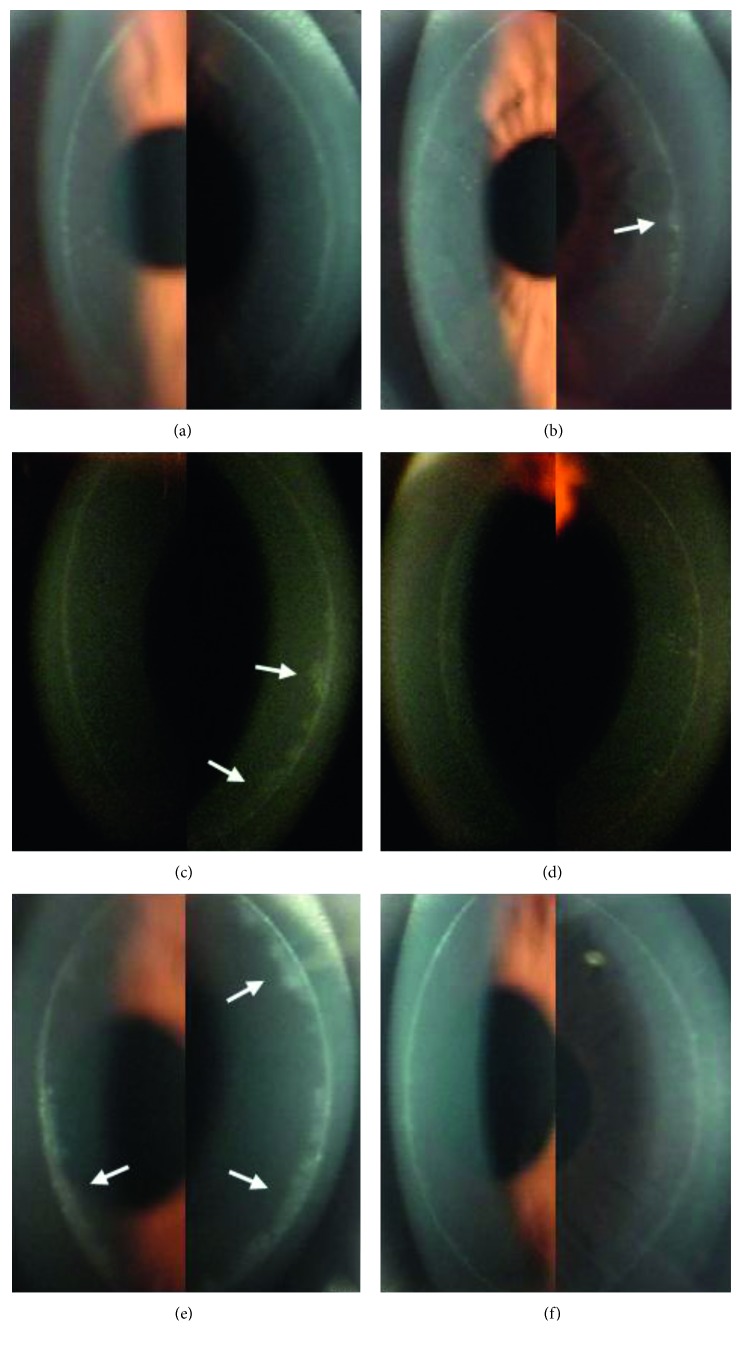
Corneal flap margin and adjacent regions photographed by slit-lamp biomicroscopy at 6 months after surgery. A narrower and less spiculated circumferential band in the BCL-treated right eye (a) and a circumferential band with a spiculated temporal side (white arrow) in the non-BCL-treated left eye (b) of a patient. A circumferential band that is wider and more spiculated on the nasal side (white arrow) in the non-BCL-treated right eye (c) and a narrower and less spiculated circumferential band in the BCL-treated left eye (d) of a patient. A circumferential band that is wide and spiculated on both the temporal and nasal sides (white arrow) in the non-BCL-treated right eye (e) and a smooth, narrow circumferential band in the BCL-treated left eye (f) of a patient.

Twenty-three (56.1%) patients had a less intense healing response at the flap margin in the BCL-treated eye than in the one without BCL treatment. Three patients had a smooth circumferential band that was wider in the eye without BCL application than in the one with BCL application (Figures 3(a) and 3(b)). Two patients had a circumferential band with a slightly more reflective focal scar in the non-BCL–treated eye than in the treated eye (Figures 3(c) and 3(d)). Five patients had a wider and more spiculated circumferential band in the untreated eye than in the BCL-treated eye across a small range of the band (Figures 4(a) and 4(b)). Eight patients had a wider and more spiculated circumferential band in the untreated eye than in the treated eye across a moderate range (Figures 4(c) and 4(d)). Five patients had a wider and more spiculated circumferential band in the untreated eye than in the treated eye in a wide range (Figures 4(e) and 4(f)).

No BCL-treated eye was found to have a greater healing response at the flap margin than the contralateral eye.

There was a statistically significant difference in healing between BCL eyes and control eyes (*P* < 0.001), revealing that BCL can significantly alleviate the fibrotic healing response of flap margins after FS-LASIK.

### 3.4. Adverse Events

No epithelial problems occurred during surgery. After surgery, one patient's control eye showed punctate epithelial defects in the central and inferior areas of the cornea the day after surgery, whereas the other eye had an intact epithelium after BCL removal. This patient received conventional medication, including preservative-free tear supplements, for several days until the epithelial defect healed. This type of epithelial defect recurred twice in this patient within the first 3 months postoperatively. After that time, no recurrence of epithelial problems was noted.

No other serious complications (e.g., epithelial ingrowth, diffuse lamellar keratitis, infection) occurred in any of the eyes that underwent surgery.

## 4. Discussion

BCLs are widely used after corneal refractive surgery, including laser-assisted subepithelial keratomileusis (LASEK) or photorefractive keratectomy, because they protect the corneal wound, prompt the corneal epithelium to heal, and relieve patients' discomfort [7–9]. They are also frequently applied to keep the flap in its proper position after LASIK and thus prevent corneal flap–related complications, such as buttonhole flaps, partial flaps, free caps, epithelial defects, and postoperative traumatic dislocation of LASIK flaps [10, 12, 13]. In the present study, we evaluated the effect of BCL application after FS-LASIK. We found that BCL significantly reduced the fibrotic wound healing response of the flap margins at 6 months after FS-LASIK. The BCL eyes had smoother, narrower, less spiculated, and less reflective circumferential bands than control eyes.

When the eye is examined by slit-lamp biomicroscopy, a more visible white band is typically observed in the corneal flap periphery after FS-LASIK than after microkeratome LASIK. In contrast to a mechanical microkeratome, a femtosecond laser creates microcavities that ablate and separate the corneal tissue; therefore, an incisional gap—lacking epithelium, basement membrane, and anterior stromal tissues—forms around the edges of the flap. Separating the flap with surgical instruments and rinsing it after ablation can cause it to increase in depth and contract in diameter. The peripheral gap may widen after flap repositioning.

In vivo corneal confocal microscopy studies have shown that epithelial cells fill the peripheral gap in the early postoperative period [3, 4]. Keratocyte transformation and stromal inflammation induced by the femtosecond laser, interaction between epithelial cells and keratocytes, and stromal infiltration of inflammatory mediators carried by tears might be involved in the corneal wound healing process, producing an intense fibrotic scarring reaction at the flap margin [2–4, 14–17].

Although the same femtosecond laser spot separation is used for flap and cap side cuts, a more visible white fibrotic scar is typically observed in the corneal flap periphery after FS-LASIK than in the cap incisional periphery after small incision lenticule extraction (SMILE). The space between the flap margins is wider in FS-LASIK-treated eyes than the space between the cap margins in SMILE-treated eyes 6 months postoperatively, and more epithelial cells fill the empty peripheral space [18]. We speculated that this may be because the concentric contraction of the flap in FS-LASIK differs from that of the cap in SMILE.

The compression function of BCLs may be the main reason why they reduced the fibrotic scarring reaction of the flap margin in the present study. It can be hypothesized that BCL compression reduces the swelling and contraction of the corneal flap, leaving a narrower empty space that fills with an epithelial plug. Fewer epithelial cells and less room for change in volume might reduce the fibrotic response. The BCL may also compress the flap to form a tighter and more stable attachment to the stromal bed and reduce stromal infiltration of tear-borne inflammatory mediators. Therefore, a less intense lamellar stromal inflammation response leads to smoother, less spiculated edges when the flap margins heal.

The present study did not reveal whether BCLs could decrease the incidence rate of epithelial ingrowth.

Although the present study showed that the BCL did not significantly decrease tearing after surgery, the lens can nevertheless act as a barrier to the entry of tear-borne inflammatory mediators, meibomian gland secretions, or foreign material into the incisional gap or lamellar stroma. In that sense, the highly reflective fibrotic scars that appeared at the flap margins of eyes that were not treated with BCLs might have been caused by some foreign material that entered the incisional gap or stromal bed, and stimulated an intense wound-healing response.

The effect of BCLs on patients' comfort after LASIK is controversial. Ahmed reported that BCL-treated eyes were more comfortable than untreated eyes for the first 3 hours after LASIK, but less comfortable than their counterparts the next morning [19]. Orucov drew the same conclusion and suggested applying BCLs immediately after LASIK, followed by removal 1 hour later [20]. Sekundo concluded that BCL application after LASIK improved postoperative comfort in less than a third of all patients; those who felt an improvement were males with a history of good contact lens tolerance and Schirmer II values over 16 mm [21]. The beneficial effects of BCLs on wound healing and patient comfort after FS-LASIK have not previously been demonstrated in a prospective randomized study. Our analysis of the patients' subjective responses indicated that the BCL eyes developed less pain and less photophobia but more foreign-body sensation than control eyes. There was no significant difference in tearing between the two groups. Many patients experienced more pain and more photophobia in their control eye than in their BCL eye in the first few hours after surgery, followed by more foreign-body sensation in the BCL eye until the lenses were removed the next day. The reason why a BCL relieves symptoms is that it protects the flap wound against mechanical irritation by eyelid rubbing after FS-LASIK just as effectively as it does after LASIK. It may also compress the flap to a more stable position and reduce the effect of shear on the eye. However, the beneficial effect of BCL in alleviating the reaction and irritation caused by the surgery was temporary and limited in the present study.

The present study also showed that visual acuity was worse in the BCL group than in the control group on the first postoperative day once the contact lenses were removed. These results are consistent with many previous studies. For example, studies by Montes and Orucov showed that corneal flap edema due to overnight BCL application contributed to poor UCVA, [20, 22] and Seguí-Crespo et al. revealed that the fitting of a silicone-hydrogel BCL after uneventful LASIK provoked morphological changes in the ocular structures that may lead to a worse UCVA outcome [23]. We suspect that the edema and irritation of the corneal epithelium caused by BCL removal also temporarily blurred the patients' vision, as an improvement in visual acuity was observed 1–2 days later, with no significant difference in refraction or visual acuity between the two groups 6 months postoperatively. Although the beneficial effect of BCLs on the wound-healing response of the flap margins did not lead to better vision, the lenses were safe for use in postoperative recovery.

There were several limitations in the present study. Owing to a lack of published uniform standards, the wound healing response of the flap margins was not quantitatively analyzed and may have been influenced by unknown factors. However, the within-subjects design of this comparative study, with each patient receiving different treatments in the right and left eyes, eliminated as many potential confounders as possible. This study was a midterm study, and further investigation is needed to better understand the long-term impact of BCLs on the wound-healing response of flap margins.

## 5. Conclusion

In this present study, eyes that were treated with BCLs after FS-LASIK surgery had less discomfort than those that were not, although this beneficial effect was temporary. BCLs also alleviated the fibrotic wound healing response at the flap margins, although this beneficial effect did not lead to better visual acuity or refraction. BCLs may merit consideration as an option for improving comfort after FS-LASIK in certain patients with good contact lens tolerance. BCLs might make FS-LASIK more perfect, and make postoperative cornea more flawless.

## Figures and Tables

**Table 1:** Baseline parameters of eyes with and without BCL application.

	Total	BCL eyes	Control eyes	*P*
Age (years)	22.5 ± 14.8 (18–33)			
Sex (F/M)	18/23			
R/L		20/21	21/20	
*Preoperative measurements*				
Mean spherical refraction (D)		−4.31 ± 1.77 (−10.25 to −1.50)	−4.44 ± 1.80 (−10.75 to −1.50)	0.244
Mean cylindrical refraction (D)		−0.36 ± 0.50 (−2.00 to 0.00)	−0.34 ± 0.51 (−2.00 to 0.00)	0.688
BCVA		1.08 ± 0.13 (1.0–1.5)	1.08 ± 0.16 (0.8–1.5)	0.799
IOP		14.9 ± 2.8 (7.7–20.4)	15.2 ± 3.1 (8.4–20.1)	0.318
CCT		536.3 ± 24.8 (500–620)	537.0 ± 25.3 (495–616)	0.513
Ablation depth		83.4 ± 18.4 (43–132)	86.3 ± 19.8 (43–145)	0.060
*Postoperative measurements*				
UCVA 1 day		0.83 ± 0.20 (0.3–1.0)	0.92 ± 0.13 (0.6–1.0)	0.032
UCVA 3 days		0.94 ± 0.08 (0.8–1.0)	0.97 ± 0.07 (0.8–1.0)	0.323
UCVA 6 months		1.07 ± 0.11 (1.0–1.5)	1.08 ± 0.12 (0.9–1.5)	0.359
BCVA 6 months		1.14 ± 0.11 (1.0–1.5)	1.14 ± 0.13 (1.0–1.5)	0.519
Spherical refraction (D) 6 months		0.01 ± 0.30 (−0.50 to 0.50)	−0.01 ± 0.30 (−0.75 to 0.50)	0.816
Cylindrical refraction (D) 6 months		−0.21 ± 0.20 (−0.50 to 0.00)	−0.17 ± 0.20 (−0.50 to 0.00)	0.291
IOP 6 months		10.2 ± 2.5 (5.5–14.7)	10.3 ± 2.5 (5.5–17.2)	0.984

Values are mean ± SEM (range). Patients underwent bilateral femtosecond laser-assisted in situ keratomileusis (FS-LASIK surgery) followed by application of a bandage contact lens (BCL) to one eye (BCL eyes) but not the other (control eyes). F/M, female/male; R/L, right/left eye; D, diopters; BCVA, best-corrected visual acuity; IOP, intraocular pressure; CCT, central corneal thickness; UCVA, uncorrected visual acuity.

**Table 2:** Self-reported symptoms after FS-LASIK with or without postoperative BCL application.

Score	Number of patients reporting		*P*
Pain	BCL eyes	Control eyes	0.041
0	22	15	
1	15	14	
2	3	10	
3	1	2	

Photophobia			0.003
0	0	0	
1	35	24	
2	6	17	
3	0	0	

Foreign-body sensation			0.001
0	8	27	
1	29	12	
2	4	2	
3	0	0	

Tears			0.118
0	14	6	
1	20	28	
2	7	6	
3	0	1	

Patients underwent bilateral femtosecond laser-assisted in situ keratomileusis (FS-LASIK surgery) followed by application of a bandage contact lens (BCL) to one eye (BCL eyes) but not the other (Control eyes). Symptom scores were self-reported: 0, not present; 1, mild; 2, moderate; 3, severe.

## Data Availability

The data used to support the findings of this study are included within the supplementary information file.

## Supplementary Materials

Supplementary Materials The data used to support the findings of this study are included within the supplementary information file.
